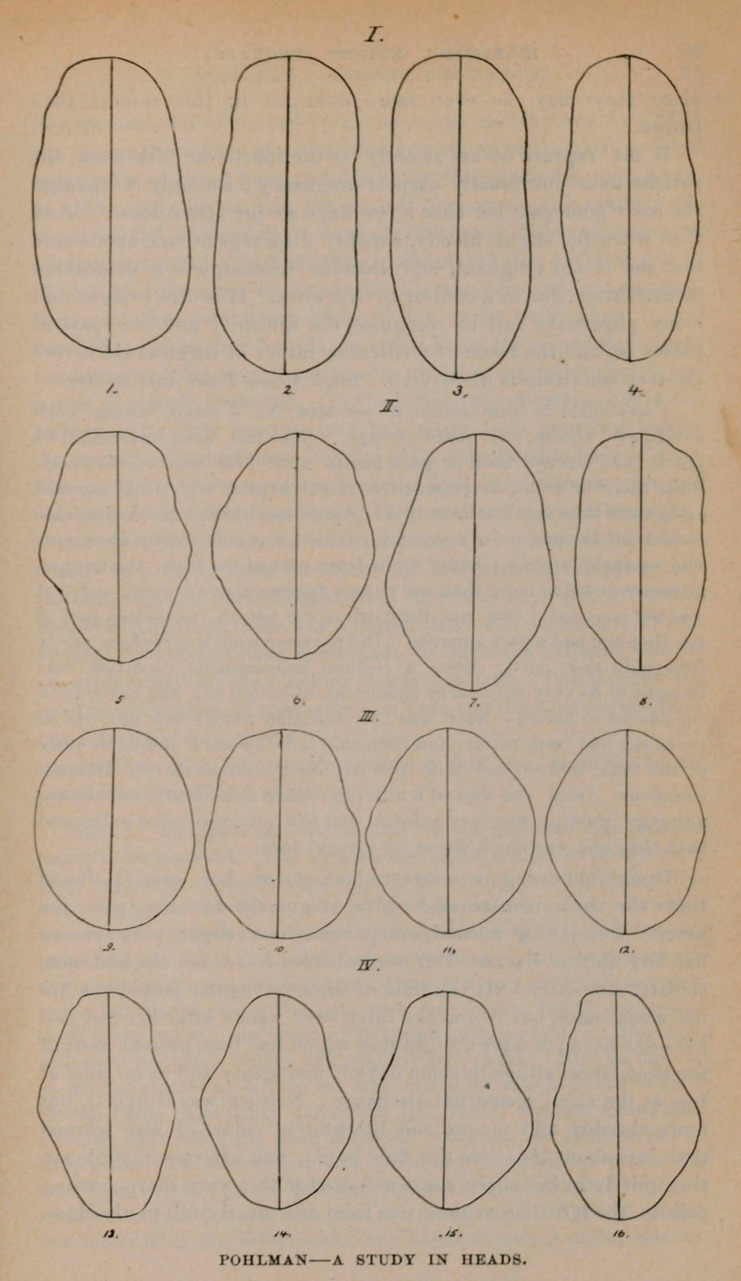# Ectopic Pregnancy

**Published:** 1895-08

**Authors:** Henry D. Ingraham

**Affiliations:** Professor of gynecology, Niagara University, Gynecologist to Buffalo Hospital of the Sisters of Charity.


### ECTOPIC PREGNANCY.

By HENRY D. INGRAHAM, M. D.,

Professor of gynecology, Niagara University, Gynecologist to Buffalo Hospital of the Sisters of Charity.

IT IS not often in ectopic pregnancy that the physician is consulted before rupture occurs. In fact, most of the patients whom I have seen do not think there is anything unusual with them until rupture happens, except that possibly they may be pregnant; yet often this is not suspected, and when rupture takes

POHLMAN—A STUDY IN HEADS.

place they may be even more doubtful in this respect than before.

If the rupture occurs as early as the fourth or fifth week, the patient does not usually suspect pregnancy; she only thinks that she has "gone past her time a few days, as she often does." And then when the slight, bloody, shreddy discharge occurs, she is sure that she is not pregnant, and that the discharge is a disordered menstruation, due to a cold or to overwork. It is also evident that many physicians fail to recognise the difficulty and the patient passes beyond the reach of medical or rather of surgical aid before the true condition is discovered. Such a case I saw last winter.

I was called in consultation to see Mrs. X., a large, strong, well-developed woman, 24 years of age. She had been married two years; had always been in good health until four weeks previously. Since then she had suffered much from pelvic pain, which was excruciating each time her bowels moved. Sometimes there was considerable abdominal distention for several days, then it would mostly disappear. She was pale, anemic; had a slight fever part of the time, the temperature never being more than one or two degrees above normal, and that was not constant. She was dizzy when she sat up, nauseated part of the time and had a poor appetite. Did not sleep well, was restless, partly from pain and partly from a restless indescribable condition, was thought to be very nervous or hysterical, although she had never been considered so before. Such was her condition when I saw her, yet at times she had been better, had been able to be up about the house some of the time, and, in fact, had been out on the street on two different occasions. Once she walked a mile, the other time nearly two miles, although walking was very painful, and she returned home exhausted each time and was much worse for several days.

Upon obtaining a correct history of her case I found that she had menstruated quite regularly for the past ten years. Everything normal, except sometimes slight pain; never had any uterine disease. Ten weeks before I saw her she had menstruated normally. At the time of the next regular period she did not menstruate, but two weeks later—six weeks after her last period—she began to have a slight flow which had been present most of the time since, although some days it was scanty and at no time as free as the usual menstrual discharge. Neither was it like it, but more shreddy and mucus, and brighter in color. I also learned that just about the time the flow began, one afternoon, while sitting quietly in her chair, she was seized with a very sharp, cutting pain in the left iliac region, was faint and nearly fell to the floor.

She was assisted to the bed by a friend who said she was very pale; for two or three days she was unable to sit up or to raise her head on account of being faint and dizzy, but this gradually wore away, as did the pain in the side, yet none of these symptoms entirely disappeared and at times they were much worse. Upon examination a boggy mass, fully half as large as the closed fist, could be felt at the left and posterior to the uterus. It was very tender and sensitive, and the patient experienced the same pain when it was touched as when her bowels moved. From the examination and the history of the case, there was no doubt in my mind that it was a case of ectopic gestation. Yet her attending physician could not believe so, chiefly because she had never been pregnant before. An operation was advised, but the patient declined to have it done. She gradually grew worse and finally died of peritonitis two and one-half weeks after I saw her. No post-mortem was allowed and hence there may be some doubt of the correctness of the diagnosis, yet, judging from the history of the case, the symptoms and physical signs, it does not seem that there was any room for doubt. But admitting that there was a doubt, there was no reason why an attempt should not have been made to save the patient's life by an exploratory incision before it was too late. The patient and her friends wished an operation less than two days before she died, but I declined to perform it.

Another case, somewhat similar to this one, occurred in the practice of Dr. Tobie, of this city:

Mrs. K., 36 years old, the mother of six children, the youngest nearly three years old. Had always been in good health and menstruated quite regularly, except when pregnant or nursing her children. She had been regular for twenty months previous to her last period, which was two weeks past due. She did her usual housework, and the day before she was taken ill she did a large washing. At three o'clock the next morning she was awakened from sleep by a very sharp, cutting pain in the left iliac region. The pain was very severe, and in attempting to sit up in bed she was faint and fell back upon her pillow. Her husand gave her some whisky and applied hot cloths. After an hour or so she felt somewhat better, but was unable to raise her head without feeling faint. The family physician, Dr. Tobie, was not called until late the next day. He gave her some medicine and ordered her to keep quiet. The following day he called and found her much as at the first visit, only free from pain and hence more comfortable. At this visit he learned that she had gone two weeks past her period, that a few hours after the severe pain, she began to have a slight, mucous, bloody discharge, which she thought was the beginning of her period, as she "had gone past her time a few days, as she often did."

Upon examination nothing abnormal could be detected in the pelvic region, except it was extremely tender, especially on the left side, but no mass or enlargement could be outlined. The doctor had in mind a ruptured tubal pregnancy, yet the symptoms were not conclusive, and as the patient was not any worse he decided to wait. In a few days he became convinced that there was some serious pelvic trouble, most probably an ectopic pregnancy with rupture of the tube. I was called in consultation and found the patient in bed, unable to sit up on account of being faint and dizzy, very pale, quick rapid pulse, temperature about 103° and abdomen considerably distended and very tender, so much so that a thorough examination was impossible. From the history of the case and the symptoms I felt confident that Dr. Tobie was correct in his diagnosis of a ruptured ectopic pregnancy, and advised that the patient be removed to the hospital for operation.

This was delayed a few days owing to the objections of friends. They, however, became satisfied that the patient could not recover without an operation, and she was removed to the Buffalo Hospital of the Sisters of Charity two weeks after the rupture occurred. After as thorough a preparation as her condition would allow, she was operated upon the second day after admission. Upon opening the abdomen a considerable mass of clotted blood was found in the left iliac region, which was beginning to break down and suppurate. This was removed and the left tube ligated and removed. The intestines were very much distended and one mass of adhesions. These were separated and broken up, as far as could be, and the abdominal cavity thoroughly washed out with hot sterilised water, a glass drainage-tube introduced and the wound closed. The patient was returned to bed in as good condition as could be expected, yet we had but little hope of her recovery. Just before the operation her temperature was 103° and pulse 120, weak and feeble.

Her condition continued about as it was before the operation until her bowels were thoroughly moved by calomel and salines the second day after the operation. This relieved the abdominal distention and the temperature fell a little—one degree. For the first day considerable bloody serum escaped through the drainage-tube, but this grew less and on the second day the tube was removed. From this time on, the patient continued about the same for a week, then began to improve. Convalescence was slow but uninterrupted, and four weeks after the operation she left the hospital perfectly well, although not strong. Since then she has continued to gain flesh and to improve in strength, and has been as well as ever. The symptoms in the latter case were not as distinct of ectopic pregnancy with rupture as in the former one, yet an examination of the removed tube showed a rupture at the middle third, and a microscopical examination of the *débris* revealed the chorionic villi.

The condition of the patient was such that the outcome of an operation was problematical, to say the least, yet the final result fully justified the attempt made to save her life.

When any woman who has menstruated regularly and who has passed her period from four to twelve weeks, is suddenly seized with pain in either iliac region; becomes faint; she may lose consciousness, but usually does not; is dizzy, nauseated, pale, generally unable to sit up, tender and sensitive over lower part of abdomen; often has a desire to go to stool and when she does is not relieved, and upon vaginal examination a boggy mass is found at one side and posterior to the uterus, and a slight, bloody, shreddy mucous discharge occurs, she has the classical symptoms of a ruptured tubal pregnancy and an exploratory incision should be made as soon as the patient can be prepared. Even if some of these symptoms should not be detected, as in the last case mentioned—no mass could be discovered on vaginal examination—an exploration should be made.

No physician is justified in allowing his patient to pass beyond assistance by waiting, or by the use of measures, the only effect of which is to lead the friends to think that something is being done.

405 Franklin Street.

## Figures and Tables

**Figure f1:**